# Rapid diagnosis of MDR and XDR tuberculosis with the MeltPro TB assay in China

**DOI:** 10.1038/srep25330

**Published:** 2016-05-06

**Authors:** Yu Pang, Haiyan Dong, Yaoju Tan, Yunfeng Deng, Xingshan Cai, Hui Jing, Hui Xia, Qiang Li, Xichao Ou, Biyi Su, Xuezheng Li, Zhiying Zhang, Junchen Li, Jiankang Zhang, Shitong Huan, Yanlin Zhao

**Affiliations:** 1 National Center for Tuberculosis Control and Prevention, Chinese Center for Disease Control and Prevention, Beijing, China; 2 PATH, Beijing, China; 3 Department of Clinical Laboratory, Guangzhou Chest Hospital, Guangdong Province, Guangzhou, China; 4 Katharine Hsu International Research Center of Human Infectious Diseases, Shandong Provincial Chest Hospital, Jinan, China; 5 Bill and Melinda Gates Foundation, China Office, Beijing, China

## Abstract

New diagnostic methods have provided a promising solution for rapid and reliable detection of drug-resistant TB strains. The aim of this study was to evaluate the performance of the MeltPro TB assay in identifying multidrug-resistant (MDR-) and extensively drug-resistant tuberculosis (XDR-TB) patients from sputum samples. The MeltPro TB assay was evaluated using sputum samples from 2057 smear-positive TB patients. Phenotypic Mycobacterial Growth Indicator Tube (MGIT) 960 drug susceptibility testing served as a reference standard. The sensitivity of the MeltPro TB assay was 94.2% for detecting resistance to rifampicin and 84.9% for detecting resistance to isoniazid. For second-line drugs, the assay showed a sensitivity of 83.3% for ofloxacin resistance, 75.0% for amikacin resistance, and 63.5% for kanamycin resistance. However, there was a significant difference for detecting kanamycin resistance between the two pilot sites in sensitivity, which was 53.2% in Guangdong and 81.5% in Shandong (*P* = 0.015). Overall, the MeltPro TB assay demonstrated good performance for the detection of MDR- and XDR-TB, with a sensitivity of 86.7% and 71.4%, respectively. The MeltPro TB assay is an excellent alternative for the detection of MDR- and XDR-TB cases in China, with high accuracy, short testing turn-around time, and low unit price compared with other tests.

Despite encouraging progress in tuberculosis (TB) control, TB remains a leading cause of morbidity and mortality worldwide^1^,^2^. The World Health Organization (WHO) estimated that there were 9 million incident TB cases and 1.5 million deaths from TB in 2013^3^. The emergence of drug-resistant TB—especially multidrug-resistant TB (MDR-TB, defined as resistance to at least isoniazid and rifampicin) and extensively drug-resistant TB (XDR-TB, defined as MDR-TB plus resistance to any fluoroquinolone and kanamycin, amikacin, or capreomycin)—is considered the greatest obstacle to global TB control due to difficulties in diagnosis and treatment^4^,^5^,^6^. Globally, in 2013, an estimated 480,000 and 43,200 people developed MDR-TB and XDR-TB, respectively. However, only 136,000 MDR-TB cases (28%) were detected and notified, and the situation for detecting XDR-TB was even more unsatisfactory because of the lack of capability to detect susceptibility against second-line drugs in many TB high-burden countries^3^. Hence, there is an urgent need to ensure that all drug-resistant TB suspects undergo testing for susceptibility to anti-TB drugs to initiate effective treatment regimens and appropriate measures of infection control^2^,^7^.

Because of the slow growth rate of *Mycobacterium tuberculosis* (MTB), conventional phenotypic drug susceptibility testing (DST) typically takes 1 to 3 months to determine the drug resistance profiles of MTB isolates^8^,^9^,^10^. More importantly, all culture-based conventional methods require a biosafety category 3 laboratory facility and extensive training of personnel, requirements that are largely unattainable in developing countries^10^.

In recent years, several commercial molecular tests have been developed to determine the drug resistance of MTB isolates based on the detection of specific genetic mutations conferring resistance^2^,^11^,^12^,^13^,^14^. Of these rapid tests, the GenoType MTBDR (Hain Lifescience, Germany) and GeneXpert (Cepheid, USA) have been endorsed by WHO for the detection of rifampicin (RIF) and/or isoniazid (INH) resistance^8^. Although most available molecular diagnostics detect resistance to RIF and INH, the choice of molecular assays to detect resistance to second-line drugs is far more limited, except for GenoType MTBDRsl from Hain Lifescience^7^. On the basis of published reports, WHO decided not to recommend this assay as a replacement for conventional DST to rule out resistance in routine clinical practice^15^. The shortage of DST technologies for second-line drugs highlights the need for the development and evaluation of new molecular tools to improve diagnosis of MDR- and XDR-TB.

The MeltPro TB assay, developed by Zeesan Biotecheh (Xiamen, China), is an innovative molecular test for detection of resistance to the main first-line and second-line anti-TB drugs^16^. Unlike commercially available molecular tests, this technology is based on melting curve analysis with dually labeled probes, which retrieves the melting temperature (*T*_m_) shift from the wide-type into the genetic mutation of MTB. The intrinsic feature of this assay makes it possible to cover two fragments (more than 20 continuous nucleotides per fragment) associated with drug resistance in one assay; it is also easy to perform without cumbersome hybridization^16^,^17^. The MeltPro TB assays for detecting RIF, INH and fluoroquinolone resistance have been officially approved by the China Food and Drug Administration (CFDA), marking them possible for clinical practice. Several previous studies found that the sensitivity and specificity of MeltPro were 93.4% and 97.4%, respectively, for RIF resistance, and 90.8% and 96.4%, respectively, for INH resistance using the proportion method on the solid medium as a reference standard^16^,^18^, suggesting that this assay could be used as an alternative to phenotypic DST. However, these studies evaluated the MeltPro TB assay on cultured isolates, and assay performance for detecting resistance to second-line drugs was not evaluated. Thus, there is a need to validate assay performance in settings with a high prevalence to evaluate the feasibility of using the test for routine detection of XDR-TB cases from clinical samples.

In this paper, we report on a multicenter study to evaluate use of the MeltPro TB assay on sputum samples for detection of resistance to first-line and second-line TB drugs. The results provide insight on the potential to scale up this new technology in China.

## Results

### Patient enrollment

**Figure 1:**
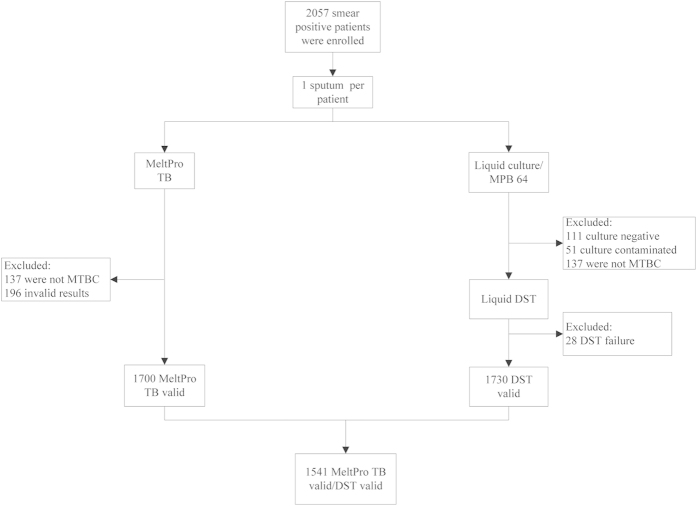
Flow diagram outlining patient enrollment and outcomes.

A total of 2057 smear-positive patients were enrolled in this evaluation. One specimen was collected from each patient and digested with NALC-NaOH for liquid DST and MeltPro TB assay. Figure 1 depicts how samples were processed and achieved. Of the specimens for liquid DST, 111 (5.4%) were negative in culture; 51 (2.5%) were contaminated; 137 (6.7%) were identified as nontuberculous mycobacteria by MPB64 monoclonal antibody assay; and the DST results for 28 (1.4%) specimens were invalid. For the MeltPro TB assay, 196 (9.5%) specimens were excluded from the study due to nontuberculous mycobacteria and invalid results. Overall, 1541 specimens were used for evaluating the performance of the MeltPro TB assay (Fig. 1).

### Performance of MeltPro TB assay for RIF and INH resistance

We first evaluated the performance of the MeltPro TB assay versus liquid DST for detection of RIF and INH resistance. As shown in Table 1, among 278 RIF-resistant TB patients diagnosed by DST, 262 cases were identified by MeltPro TB assay, with a sensitivity of 94.2% (see Table 1 for confidence intervals). In addition, 1231 out of 1263 RIF-susceptible TB patients diagnosed by DST were confirmed by MeltPro TB assay, indicating a specificity of 97.5%. For INH resistance, the sensitivity and specificity of the MeltPro TB assay were 84.9% and 98.0%, respectively (Table 2). We also calculated MeltPro TB assay performance for MDR-TB detection measured against liquid DST. Overall, the sensitivity for MDR-TB was 86.7%, and the specificity was 97.7% (Table 3).

### Performance of MeltPro TB assay for detecting resistance to second-line drugs

In comparison with conventional liquid DST as the gold standard, the MeltPro TB assay showed a sensitivity of 83.3% for OFLX, 75.0% for AMK, and 63.5% for KAN (Tables 4, 5, 6). The specificity for detecting resistance to all three drugs was greater than 98%: 98.1% for OFLX, 98.7% for AMK, and 99.2% for KAN. For OFLX and AMK, sensitivity was similar in both Shandong and Guangzhou. For KAN, however, there was a significant difference between the two pilot sites in sensitivity, which was 53.2% in Guangdong and 81.5% in Shandong (*P* = 0.015).

In view of the significant different performance of MeltPro for detecting KAN resistance between Shandong and Guangdong, the gene mutations of 74 phenotypically KAN-resistant strains were analyzed by DNA sequencing. As shown in Table 7, the mutation located in 1401 A→G of the *rrs* region was identified as the most frequent mutation conferring KAN resistance in both Shangdong (20/27, 74.1%) and Guangdong (19/47, 40.4%). In contrast, we also observed that there were 5 (18.5%) and 21 (44.7%) KAN-resistant isolates harboring no mutation in Shangdong and Guangdong, respectively. Statistical analysis revealed that there was a significantly higher proportion of KAN-resistant isolates with genetic mutations in Shandong in comparison with that in Guangdong (*P* = 0.023).

We also analyzed the sensitivity and specificity of the MeltPro TB assay for diagnosing XDR-TB cases. Overall, the accuracy of the MeltPro TB assay for detecting XDR-TB was 98.8%, with a sensitivity and specificity of 71.4% and 99.6%, respectively (Table 8).

## Discussion

We performed the first multicenter study to access the diagnostic accuracy of the MeltPro TB assay for detection of MDR- and XDR-TB cases from clinical sputum samples. The assay demonstrated satisfactory sensitivity and specificity for drug-resistant *M. tuberculosis* among patients at hospitals with high burden of drug-resistant TB, especially for RIF and INH.

Several commercial diagnostic tools for detection of drug-resistant TB have been evaluated in laboratories of various types in China. These tools have included Genotype MTBDR from Hain, Genechip from CapitalBio, and GeneXpert from Cephid^8^,^19^,^20^. The sensitivities of these molecular assays for detection of RIF resistance have varied between 87.10% for GeneXpert^20^ and 88.3% for Genotype MTBDR^19^—lower than the sensitivity of 94.2% in this study. Several factors may be responsible for the difference. First, the detection of heteroresistance by the MeltPro assay is the major contributor to the increased sensitivity for detection of RIF resistance. Because of the inherent limitations of the interpretation system for Genechip, it is difficult to detect less than 50% RIF heteroresistance. For GeneXpert, the presence of susceptible bacteria would result in the occurrence of amplification curves, thereby resulting in missed detection of RIF heteroresistance. A prior study has demonstrated that a high melting curve assay can detect the presence of RIF resistance mutations down to a concentration of 5% mutant DNA^21^. Hence, given the high prevalence of heteroresistance in China^22^,^23^, the better capability of MeltPro to detect mixed infection may help laboratory staff identify more RIF-heteroresistant TB cases. Second, the different phenotypic DST methods used may be another explanation for the difference.

The sensitivity of MeltPro for INH resistance was also higher than that for the other evaluated methods. A recent molecular epidemiological study from China has demonstrated that the combination mutations in *katG* gene and the promoter of *inhA* gene can only identify about 75% of INH-resistant isolates, while the nucleotide substitutions in the intergenic region of *oxyR-ahpC* rather than other mutations confers 5.1% of INH resistance in the MDR population of China^24^. The inclusion of the *ahpC* promoter region therefore increased the test sensitivity (84.9%) of MeltPro to detect the INH resistance when compared with 80.34% for Genechip and 80.2% for Genotype MTBDR^8^,^19^.

In the current study, the MeltPro assay reliably detected OFLX resistance, with a sensitivity of 83.3% and a specificity of 98.1%. Unlike the situation with tests for RIF and INH resistance, the diagnostic accuracy of molecular tools shows significant heterogeneity for detection of fluoroquinolone (FQ) resistance across studies, varying from 68% to 92%^15^. On one hand, the frequencies of mutations conferring FQ resistance differ from one geographic region to another. For example, one study found that 83% of FQ-resistant TB isolates from Russia harbored *gyrA* mutations^25^, whereas this percentage in Taiwan was only 50%^26^. Hence, variation in the molecular characteristics of FQ-resistant isolates from region to region may be the primary reason for differences in diagnostic accuracy. On the other hand, heteroresistance is considered to be an important mechanism for the emergence of resistance, and a high rate of heteroresistance is associated with settings with a high TB prevalence^22^. In a recent study, Zhang and colleagues found FQ heteroresistance in 23% of TB isolates in China^22^, which is a significantly higher percentage than has been observed in South Korea and the United States, with a proportion of 9%^21^,^22^. Hence, in light of the poor sensitivity of molecular methods for detecting heteroresistance in comparison with phenotypic DST methods^27^, the relatively common occurrence of FQ heteroresistance may be attributed to variable performance of molecular methods for identifying FQ resistance from clinical samples.

In regard to second-line injectable drugs, the MeltPro assay showed moderate test sensitivity and high specificity for the detection of AMK resistance. By contrast, its sensitivity for predicting KAN resistance was unsatisfactory. The KAN-resistant strains isolated from Shandong are more likely to harbor genetic mutations located in the *rrs* gene and *eis* promoter than those from Guanzhou, resulting in higher sensitivity for the detection of KAN resistance. Our findings were in line with previous observations of significant variability in the sensitivity for the detection of KAN resistance across studies, ranging from 25.0% to 100.0%^15^. The different distribution of mutations with geographic origin provides a potential explanation for this heterogeneity. In line with our findings, several previous reports have demonstrated that the frequencies of mutants conferring KAN resistance show significant diversity among different regions of China, ranging from 54% in Chongqing to 100% in Hunan^28^,^29^. Hence, our findings suggest that the MeltPro assay can be used as an alternative for detecting OFLX and AMK resistance in China, although its feasibility for detecting KAN resistance is questionable. This indicates an urgent need for further studies in different settings of China.

According to the 12^th^national five-year plan drawn up by the government of China, rapid drug susceptibility testing tools will be adopted overall in prefectural and municipal TB laboratories by 2015. To provide more detailed evidence for laboratory technicians, this paper compares the four available rapid DST methods (Table 9). First, based on our evaluation, the MeltPro assay shows the favorable performance for detecting RIF and INH resistance. Second, in regard to the simplicity of operation, GeneXpert is no doubt the most automatic platform for rapid identification of RIF resistance. Because of the application of automated nucleic acid extraction devices and an in-tube detection system, the MeltPro is more convenient than Genechip and GenoType MTBDR and offers a shorter turn-around time to generate diagnostic results. Third, unlike the three other available assays, the MeltPro uses a conventional real-time PCR platform rather than any special equipment to perform clinical analysis, which is more suitable for TB laboratories in poor regions. Finally, the MeltPro assay provides the lowest reagent price for detecting RIF and INH resistance—half that of GeneXpert. Hence, the MeltPro assay is a cost-effective method for MDR- and XDR-TB diagnosis, compared to both conventional DST methods and other commercial molecular kits available in China. It has important potential for improving diagnosis and control of drug-resistant TB.

There were several obvious limitations in this study. First, several literatures have revealed that liquid DST is prone to miss some low-level resistant MTB when compared with conventional solid DST method^30^. Hence, the gold standard used in this study may influence the analytical sensitivity and specificity of MeltPro. Second, whole-genome sequencing (WGS) has been considered as a useful tool for validating mutations and confirming molecular resistance compared to phenotypic DST^7^,^31^. However, due to the high price of WGS and the large sample size, WGS was not performed in the present study. Nevertheless, our evaluation provides important evidence for further implementation of MeltPro in the diagnosis algorithm of China.

In conclusion, our data demonstrate that the MeltPro assay is an excellent method for the detection of MDR- and XDR-TB cases in China. It outperforms several commercial assays in that it provides satisfactory accuracy, short testing turn-around time, and low unit price. Because we found significant heterogeneity for the detection of KAN resistance across regions, an effective evaluation of MeltPro needs to be conducted prior to scaling this assay for detecting KAN resistance in different regions of China. Further evaluation will also be needed to investigate the impact of the MeltPro assay on patient and TB program outcomes. Wide spread use of the assay may potentially lead to faster initiation of appropriate treatment for drug-resistant TB patients as well as reduced transmission of drug-resistant TB in the community.

## Methods

### Study sites and population

This study was approved by PATH and the Ethics Committee of Chinese Center for Disease Control and Prevention. The methods used in this study were in accordance with the approved guidelines. All patients enrolled in this study provided written informed consent for the samples collected for the research study protocol.

Between January 1, 2014, and February 28, 2015, the performance of MeltPro TB assay was evaluated in two TB-specialized hospitals in China: Guangzhou Chest Hospital and Shandong Chest Hospital. All smear-positive TB patients seeking health care in these two hospitals were enrolled consecutively in the study, irrespective of co-morbidities or HIV-status. We collected one sputum specimen of more than 2 mL from each patient for further smear microscopy, liquid culture, and MeltPro TB assay.

### Smear and phenotypic DST

Direct smear was performed using light-emitting diode fluorescence microscopy for acid fast bacilli (AFB). Smears were graded according to national guidelines established by China Center for Disease Control and Prevention, which starts with negative to scanty to 4+^8^. A smear-positive specimen was digested by using the N-acetyl-L-cysteine (NALC)-NaOH method for 15 minutes, and then neutralized with sterile phosphate buffer (PBS, pH = 7.0). After centrifugation at 3,000 × g for 15 minutes, the pellet was resuspended in 2 mL PBS buffer. A 0.5 mL proportion of the decontaminated specimens was cultured on Mycobacterial Growth Indicator Tubes (MGIT, Becton Dickinson, USA). Positive cultures were confirmed as mycobacteria with Ziehl-Neelsen staining. Further species identification was performed using a commercial MPB64 monoclonal antibody assay (Genesis, Hangzhou, China). Indirect drug susceptibility of the culture-positive isolates identified as MTB was detected by the Bactec MGIT 960 automated system according to the manufacturer’s instructions. The critical concentrations were 1.0 μg/mL for rifampicin (RIF), 0.1 μg/ml for isoniazid (INH), 2 μg/mL for ofloxacin(OFLX), 0.5 μg/mL for amikacin (AMK), and 1.0 μg/mL for kanamycin (KAN)^27^,^32^,^33^. The clinical TB laboratories enrolled in this study have passed the proficiency testing for conventional DST organized by the National TB Reference Laboratory of the China CDC.

### MeltPro TB assay

MeltPro TB assay testing was conducted following the manufacturer’s instructions. Briefly, the crude DNA was extracted from a 1.0 mL aliquot of the decontaminated specimens with an automatic DNA extraction machine (Zeesan Biotecheh, Xiamen, China) using the paramagnetic particle method. Five microliters of the genomic DNA was applied to the amplification in each tube. The PCR mixture was performed in the LightCycler 480 system (Roche Applied Science, Indianapolis, USA) according to the following protocol: 2 min of decontamination at 50 °C using uracil-N-glycosylase; 10 min of denaturation at 95 °C; a 10 cycles touchdown program containing 10 s at 95 °C, 15 s at 71 °C (−1 °C/cycle), and 15 s at 78 °C; and 45 cycles of 10 s at 95 °C, 15 s at 61 °C, and 15 s at 78 °C. Melting curve analysis was started with 2 min of denaturation at 95 °C, 2 min of hybridization at 40 °C, and a stepwise increasing temperature from 40 °C to 85 °C at 1 °C/step with a 5-s stop between each step. The fluorescent signal intensity was collected at FAM and TET channels. Tm calling analysis was performed by identifying the peaks of the melting curves. An invalid result was defined as the strain with invalid result of any drug susceptibility.

### DNA sequencing

The crude genomic DNA was extracted from freshly cultured bacteria following the method described previously^34^. The genomic DNA was used as template to carry out PCR amplification. The fragments of genes conferring KAN resistance, including *rrs* and *eis* promoter, were amplified, and then sent to Qingke Company for sequencing service. DNA sequences were aligned with the homologous sequences of *M. tuberculosis* H37Rv strain (http://www.ncbi.nlm.nih.gov/BLAST).

### Data analysis

Conventional liquid DST was used as the reference standard to calculate the sensitivity, specificity, positive predictive value (PPV), and negative predictive value (NPV) of the MeltPro TB assay. All the data were entered into SPSS15.0 software as a database (SPSS Inc., USA). A chi-square test was used for statistical analysis. If the *P* value was less than 0.05, the difference was judged as significant.

## Additional Information

**How to cite this article**: Pang, Y. *et al.* Rapid diagnosis of MDR and XDR tuberculosis with the MeltPro TB assay in China. *Sci. Rep.* **6**, 25330; doi: 10.1038/srep25330 (2016).

## Figures and Tables

**Table 1:** Performance of MeltPro for detecting rifampin-resistance.

Pilot	MeltPro	DST		Total	Sensitivity (%, 95% CI)	Specificity (%, 95% CI)	PPV (%, 95% CI)	NPV (%, 95% CI)
		R	S					
Shandong	R	107	15	122	96.4(91.1–98.6)	97.6(96.0–98.2)	87.7(80.7–92.4)	99.2(98.2–99.7)
	S	4	601	605				
	Total	111	616	727				
Guangzhou	R	155	17	172	92.8(87.9–95.8)	97.4(95.8–98.4)	90.1(84.7–93.7)	98.2(96.8–99.0)
	S	12	630	642				
	Total	167	647	814				
Total	R	262	32	294	94.2(90.9–96.4)	97.5(96.4–98.2)	89.1(85.0–92.2)	98.7(97.9–99.2)
	S	16	1231	1247				
	Total	278	1263	1541				

Abbreviations: DST = drug susceptibility test, R = resistant, S = sensitive, PPV = positive predictive value, NPV = negative predictive value.

**Table 2:** Performance of MeltPro for detecting isoniazid-resistance.

Pilot	MeltPro	DST		Total	Sensitivity (%, 95% CI)	Specificity (%, 95% CI)	PPV (%, 95% CI)	NPV (%, 95% CI)
		R	S					
Shandong	R	134	13	147	86.4(80.2–91.0)	97.7(96.2–98.7)	91.2(85.5–94.8)	96.4(94.5–97.6)
	S	21	559	580				
	Total	155	572	727				
Guangzhou	R	197	10	207	83.8(78.6–88.0)	98.3(96.8–99.1)	95.2(91.3–97.4)	93.7(91.5–95.4)
	S	38	569	607				
	Total	235	579	814				
Total	R	331	23	354	84.9(81.0–88.1)	98.0(97.0–98.7)	93.5(90.4–95.6)	95.0(93.6–96.1)
	S	59	1128	1187				
	Total	390	1151	1541				

**Table 3:** Performance of MeltPro for detecting multi-drug resistance.

Pilot	MeltPro	DST		Total	Sensitivity (%, 95% CI)	Specificity (%, 95% CI)	PPV (%, 95% CI)	NPV (%, 95% CI)
		R	S					
Shandong	R	85	14	99	87.6(79.6–92.8)	97.8(96.3–98.7)	85.9(77.6–91.4)	98.1(96.7–98.9)
	S	12	616	628				
	Total	97	630	727				
Guangzhou	R	130	16	146	86.1(79. 7–90.7)	97.6(96.1–98.5)	89.0(82.9–93.1)	96.9(95.2–97.9)
	S	21	647	668				
	Total	151	663	814				
Total	R	215	30	245	86.7(81.9–90.4)	97.7(96.7–98.4)	87.8(83.1–91.3)	97.4(96.4–98.2)
	S	33	1263	1296				
	Total	248	1293	1541				

**Table 4:** Performance of MeltPro for detecting ofloxcin-resistance.

Pilot	MeltPro	DST		Total	Sensitivity (%, 95% CI)	Specificity (%, 95% CI)	PPV (%, 95% CI)	NPV (%, 95% CI)
		R	S					
Shandong	R	99	18	117	86.1(78.6–91.2)	97.1(95.4–98.1)	84.6(77.0–90.0)	97.4(95.8–98.4)
	S	16	594	610				
	Total	115	612	727				
Guangzhou	R	106	7	113	80.9(73.3–86.7)	99.0(97.9–99.5)	93.8(87.8–97.0)	96.4(94.8–97.6)
	S	25	676	701				
	Total	131	683	814				
Total	R	205	25	230	83.3(78.2–87.5)	98.1(97.2–98.7)	89.1(84.4–92.5)	96.9(95.8–97.7)
	S	41	1270	1311				
	Total	246	1295	1541				

**Table 5:** Performance of MeltPro for detecting amikacin-resistance.

Pilot	MeltPro	DST		Total	Sensitivity (%, 95% CI)	Specificity (%, 95% CI)	PPV (%, 95% CI)	NPV (%, 95% CI)
		R	S					
Shandong	R	19	8	27	76.0(56.6–88.5)	98.9(97.8–99.4)	70.4(51.5–84.2)	99.1(98.1–99.6)
	S	6	694	700				
	Total	25	702	727				
Guangzhou	R	20	12	32	74.1(55.3–86.8)	98.5(97.4–99.1)	62.5(45.2–77.1)	99.1(98.2–99.6)
	S	7	775	782				
	Total	27	787	814				
Total	R	39	20	59	75.0(61.8–84.8)	98.7(97.9–99.1)	66.1(53.4–76.9)	99.1(98.5–99.5)
	S	13	1469	1482				
	Total	52	1489	1541				

**Table 6:** Performance of MeltPro for detecting kanamycin-resistance.

Pilot	MeltPro	DST		Total	Sensitivity (%, 95% CI)	Specificity (%, 95% CI)	PPV (%, 95% CI)	NPV (%, 95% CI)
		R	S					
Shandong	R	22	5	27	81.5(63.3–91.8)	99.3(98.3–99.7)	81.5(63.3–91.8)	99.3(98.3–99.7)
	S	5	695	700				
	Total	27	700	727				
Guangzhou	R	25	7	32	53.2(39.2–66.7)	99.1(98.1–99.6)	78.1(61.2–89.0)	97.2(95.8–98.1)
	S	22	760	782				
	Total	47	767	814				
Total	R	47	12	59	63.5(52.1–73.6)	99.2(98.6–99.5)	79.7(67.7–88.0)	98.2(97.4–98.7)
	S	27	1455	1482				
	Total	74	1467	1541				

**Table 7:** Detection of mutations conferring KAN resistance by DNA sequencing in MTB isolates.

Locus	Mutation type	No. of isolates (%)		
		Shandong	Guangdong	Total
*rrs*	A1401G	20(74.1)	19(40.4)	39(52.7)
	C1402T	0(0.0)	2(4.3)	2(2.7)
	G1484T	0(0.0)	1(2.1)	1(1.4)
*eis*	−10G→→A	2(7.4)	2(4.3)	4(5.4)
	−12C→→T	0(0.0)	1(2.1)	1(1.4)
	−14G→→T	0(0.0)	1(2.1)	1(1.4)
	None	5(18.5)	21(44.7)	26(35.1)
	Total	27(100.0)	47(100.0)	74(100.0)

^a^Statistical analysis is performed to compare the proportion of KAN-resistant isolates harboring genetic mutation between Shandong and Guangdong (*χ*^2^ = 5.13, *P* = 0.023).

**Table 8:** Performance of MeltPro for detecting extensive drug-resistance.

Pilot	MeltPro	DST		Total	Sensitivity (%, 95% CI)	Specificity (%, 95% CI)	PPV (%, 95% CI)	NPV (%, 95% CI)
		R	S					
Shandong	R	16	3	19	88.9(67.2–96.9)	99.6(98.8–99.8)	84.2(62.4–94.5)	99.7(99.0–99.9)
	S	2	706	708				
	Total	18	709	727				
Guangzhou	R	14	3	17	58.3(38.8–75.5)	99.6(98.9–99.9)	82.4(59.0–93.8)	98.8(97.7–99.3)
	S	10	787	797				
	Total	24	790	814				
Total	R	30	6	36	71.4(56.4–82.8)	99.6(99.1–99.8)	83.3(68.1–92.1)	99.2(98.6–99.5)
	S	12	1493	1505				
	Total	42	1499	1541				

**Table 9:** Comparison of the performance and cost of various molecular diagnostic tools.

Diagnostic tools	Reference	Target sequence(s)	Performance^a^				Turnaround time (hours)	Need for special equipment	Cost per kit (USD)^b^
			RIF		INH				
			Sensitivity (%)	Specificity(%)	Sensitivity(%)	Specificity(%)			
GeneXpert	35	RIF: *rpoB*	95	98	NA	NA	2.5	Yes	45
Genechip	8	RIF: *rpoB*; INH: *katG*, *inhA* promoter	87.6	98.0	80.3	95.8	6	Yes	30
GenoType MTBDR V1.0	36	RIF: *rpoB*; INH: *katG*, *inhA* promoter	97.1	97.1	94.4	96.4	6	Yes	30
GenoType MTBDR V2.0	36	RIF: *rpoB*; INH: *katG*, *inhA* promoter	98.2	97.8	95.4	98.9	6	Yes	NA
MeltPro	Data from this study	RIF: *rpoB*; INH: *katG*, *inhA*, *oxyR-ahpC intergenic region*, *inhA* promoter	94.2	97.5	84.9	98.0	3.5	No	25

^a^RIF: rifampicin; INH: isonizid; NA: not available.

^b^Costs are calculated according to the market price for clinical practice in China.
